# Impact of CAD/CAM Material Thickness and Translucency on the Polymerization of Dual-Cure Resin Cement in Endocrowns

**DOI:** 10.3390/polym16050661

**Published:** 2024-02-29

**Authors:** Soshi Ikemoto, Yuya Komagata, Shinji Yoshii, Chihiro Masaki, Ryuji Hosokawa, Hiroshi Ikeda

**Affiliations:** 1 Division of Biomaterials, Kyushu Dental University, Kitakyushu 803-8580, Japan; r19ikemoto@fa.kyu-dent.ac.jp (S.I.); pty_pty_kjm@yahoo.co.jp (Y.K.); 2 Division of Oral Reconstruction and Rehabilitation, Kyushu Dental University, Kitakyushu 803-8580, Japan; masaki@kyu-dent.ac.jp (C.M.);; 3 Division of Promoting Learning Design Education, Kyushu Dental University, Kitakyushu 803-8580, Japan

**Keywords:** dental materials, luting agent, endocrown, CAD/CAM, ceramic, polymer-infiltrated ceramic network, resin composite, polymerization

## Abstract

The objective of this study is to evaluate the impact of the thickness and translucency of various computer-aided design/computer-aided manufacturing (CAD/CAM) materials on the polymerization of dual-cure resin cement in endocrown restorations. Three commercially available CAD/CAM materials—lithium disilicate glass (e.max CAD), resin composite (CERASMART), and a polymer-infiltrated ceramic network (ENAMIC)—were cut into plates with five different thicknesses (1.5, 3.5, 5.5, 7.5, and 9.5 mm) in both high-translucency (HT) and low-translucency (LT) grades. Panavia V5, a commercial dual-cure resin cement, was polymerized through each plate by light irradiation. Post-polymerization treatment was performed by aging at 37 °C for 24 h under light-shielding conditions. The degree of conversion and Vickers hardness measurements were used to characterize the polymerization of the cement. The findings revealed a significant decrease in both the degree of conversion and Vickers hardness with increasing thickness across all CAD/CAM materials. Notably, while the differences in the degree of conversion and Vickers hardness between the HT and LT grades of each material were significant immediately after photoirradiation, these differences became smaller after post-polymerization treatment. Significant differences were observed between samples with a 1.5 mm thickness (conventional crowns) and those with a 5.5 mm or greater thickness (endocrowns), even after post-polymerization treatment. These results suggest that dual-cure resin cement in endocrown restorations undergoes insufficient polymerization.

## 1. Introduction

An endocrown is a specialized dental restoration primarily utilized in dentistry for teeth that have experienced substantial damage, typically due to decay, following root canal treatment [1]. Unlike conventional crowns that envelope the entire visible portion of a tooth, endocrowns are custom-designed for molars and premolars, offering a modern and less-invasive alternative in restorative dentistry [2]. Endocrowns are inserted into the pulp chamber previously occupied by the pulp of the removed tooth and cover the occlusal surface of the tooth [3]. Unlike conventional crowns that often require additional dental posts, endocrowns bond directly to the tooth cavity, resulting in minimal removal of the natural tooth structure. A primary benefit of this conservative approach to restoration is that it dispenses with the need for extensive tooth reshaping, often required for conventional crown fitting, thereby conserving more of the natural tooth structure than conventional crowns [4]. The conservation of tooth is advantageous for the long-term health and structural integrity of the tooth. Additionally, the procedure for placing an endocrown tends to be simpler and more expedient than that for placing a conventional crown [5]. Endocrowns exhibit excellent mechanical strength and durability, particularly those fabricated from advanced ceramic materials and resin composites [6], which makes them notably effective in restoring the function and aesthetics of damaged molars and premolars.

The history of endocrowns as a dentistry concept is relatively recent compared to that of other dental restorations, such as fillings or conventional crowns. Their development and popularity are closely linked to the advancements in dental materials and adhesive technologies [6,7]. The late 20th century saw notable progress in adhesive dentistry, enabling more conservative restorations that effectively bonded to tooth structures [8]. During the period of emergence, this concept was not initially defined as the endocrown. The term “endocrown” and its unique approach gained prominence in the 1990s, aided by the introduction of high-strength ceramic materials and improved adhesive techniques [9]. The “monoblock technique”, proposed by Pissis in 1995 [10], was a precursor to endocrown restoration. A significant milestone was a 1999 publication in which Bindle and Mörmann [11] introduced the “endocrown” as a monolithic ceramic restoration, made from computer-aided design/computer-aided manufacturing (CAD/CAM) ceramic, for post-root canal treatment. In the 2000–2010s [1,12,13], the use of endocrowns expanded, supported by further enhancements in luting agents, ceramic materials, and CAD/CAM technology, leading to more precise and aesthetically pleasing restorations. Recently, endocrowns have become the favored choice for restoring endodontically treated teeth, particularly in cases of substantial tooth structure loss [3].

The clinical outcomes of endocrowns, as reported by various studies, have generally been positive, demonstrating their efficacy as a restorative option for endodontically treated teeth. Many studies have reported high survival rates for endocrowns, often comparable to conventional crowns, typically exceeding 90% over five years [14]. The clinical failure of an endocrown, particularly debonding, is an important consideration in restorative dentistry [14,15]. Factors contributing to the debonding failure of endocrowns include tooth design and preparation. If the endocrown fits poorly or errors occur during tooth preparation (e.g., undercuts or insufficient decay removal), then it can result in weak bonding and failure. The success of endocrowns also relies heavily on the correct application of adhesive techniques. The inappropriate use of luting agents, improper etching, or contamination of the bonding surface (such as by saliva or blood) during the procedure can compromise the bond strength. In addition, luting agents play a crucial role in bonding endocrowns to the remaining tooth structure [16,17]. Among the luting agents, resin-based cements are considered the optimal choice for endocrowns due to their superior bonding strength and durability [18]. Dual-cure resin cements, in particular, are favored for their versatility and reliable performance, making them highly suitable for bonding endocrowns. Their broad acceptance among dentists stems from their ability to cure through both light and chemical means, accommodating a variety of clinical scenarios. However, the polymerization behavior of dual-cure resin cements in endocrown restorations remains to be fully understood. Given the increased thickness of endocrowns compared to conventional crowns, the penetration of light into the dual-cure resin cement poses a challenge, leading to concerns regarding the efficiency of photopolymerization.

Several in vitro studies have explored the polymerization behavior of light-cure and dual-cure resin cements, as well as restorative resin composites, in endocrown restorations. Gregor et al. assessed the degree of polymerization of a light-cure restorative resin composite and dual-cure resin cement under 7.5 mm-thick endocrowns made from resin composite or ceramic using Vickers hardness measurements. Their findings indicated that the Vickers hardness values of both the dual-cure resin cement and the light-cure resin composite, when irradiated for 3 × 90 s at high irradiance, reached at least 80% of the control values [19]. Daher et al. evaluated the minimal irradiation time required to achieve sufficient polymerization of a light-cure restorative resin composite under 9.5 mm-thick endocrowns [20]. The study concluded that 40 s light curing per site (buccal, palatal, and occlusal) achieved a hardness of up to 80% of the positive control sample, indicating adequate polymerization. Kuijper et al. investigated the effects of ceramic translucency and restoration type (onlays and endocrowns) on the polymerization efficiency of dual-cure resin cement and a restorative resin composite using a high-power light device [21]. Their results demonstrated that material translucency influenced the polymerization of the resin composite under endocrowns, while the polymerization of the resin cement was not significantly affected by either translucency or restoration type.

While previous studies offer valuable insights into the use of dual-cure resin cements for bonding endocrowns, questions persist regarding the impacts of material type, thickness, and translucency on dual-cure resin cement polymerization. Therefore, this study aims to elucidate the impact of the thickness and translucency of aesthetic CAD/CAM materials on dual-cure resin cement polymerization. The first null hypothesis posits that the thickness of CAD/CAM materials does not affect dual-cure resin cement polymerization, while the second null hypothesis suggests that material translucency has no impact on the polymerization process. The materials chosen for endocrown fabrication include lithium disilicate glass, polymer-infiltrated ceramics, and resin composites. Both high-translucency (HT) and low-translucency (LT) grades of each material were selected to evaluate their respective influences on the polymerization process.

## 2. Materials and Methods

### 2.1. Materials

**Figure 1:**
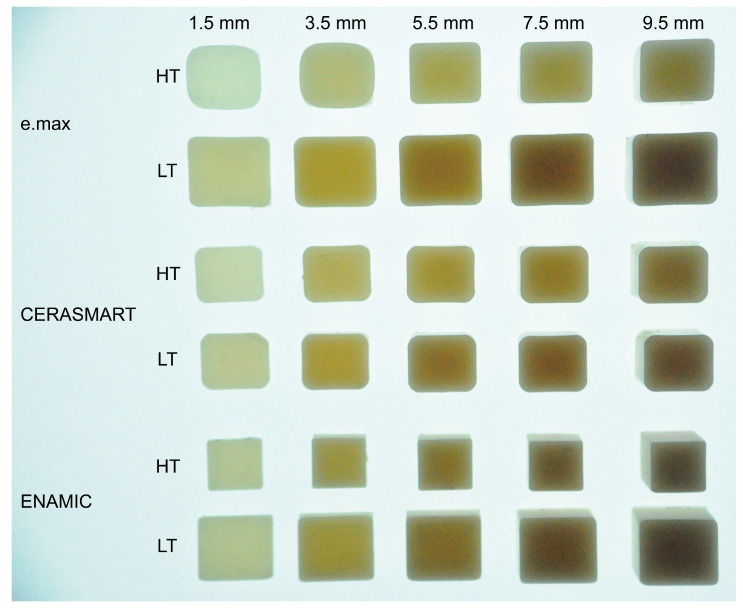
Photograph of CAD/CAM material plates showcasing varying thicknesses with high-translucency (HT) and low-translucency (LT) grades. Illumination from beneath the samples highlights the distinctions in thickness and translucency between the grades of each material.

Table 1 lists CAD/CAM materials. This study used three types of commercially available aesthetic CAD/CAM materials: lithium disilicate glass (e.max CAD), resin composite (CERASMART), and polymer-infiltrated ceramic network (ENAMIC). Both HT and LT grades were used for each material, which makes a total of six types of CAD/CAM materials. Each CAD/CAM material block was cut into plates of different thicknesses (1.5, 3.5, 5.5, 7.5, and 9.5 mm) using a diamond wheel saw (MINICUT 40, SCAN-DIA GmbH & CO. KG, Hagen, Germany) under water cooling. The surface of each plate was polished using emery papers in the order of #400, #800, and #1500. To ensure that the specified thickness was within ±0.1 mm, the resultant plate thickness was measured using a digital vernier caliper (Mitutoyo CD-15CPX, Mitutoyo Corp., Kawasaki, Japan). The polished plates were then cleaned via ultrasonication in distilled water for 5 min to eliminate debris. The resultant plates were used for the polymerization experiment of the dual-cure resin cement. Figure 1 illustrates the CAD/CAM material plates, showcasing variations in thickness along with the HT and LT grades.

Panavia V5 (Kuraray Noritake Dental, Inc., Tokyo, Japan) was used as the dual-cure resin cement.

### 2.2. Photopolymerization of Dual-Cure Resin Cement and Post-Polymerization Treatment by Aging

**Figure 2:**
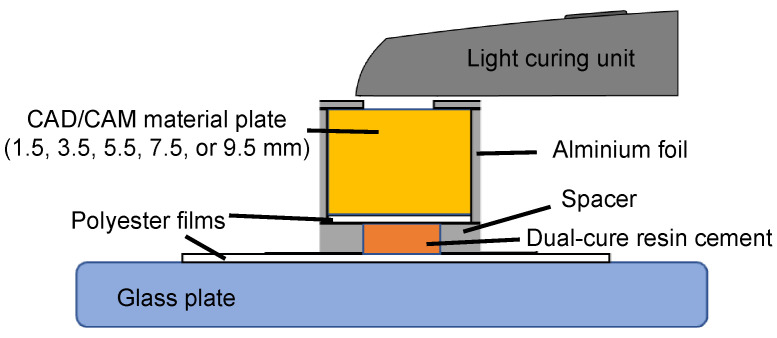
Schematic view of experimental setup for photopolymerization of dual-cure resin cement through CAD/CAM material plate.

Photopolymerization of the dual-cure resin cement was conducted by light irradiation through a CAD/CAM material plate, as depicted in Figure 2. In the experimental setup, the dual-cure resin cement was placed between two 25 μm polyester films (Lumirror film T60, Toray Industries, Inc., Tokyo, Japan) that served as the top and base layers. To ensure a standardized thickness of the dual-cure resin cement, a 1 mm-thick spacer was positioned adjacent to the resin cement. This assembly was placed on a glass plate and a CAD/CAM material plate was mounted on the sandwiched cement. Light irradiation was performed using a handheld light-curing unit (VALO GRAND; Ultradent Products Inc., South Jordan, UT, USA) placed in direct contact with the CAD/CAM material plate. Continuous light irradiation was performed for 90 s at a temperature of 25 °C, using the light-curing unit in high-power-plus mode (1600 w/cm^2^). The dual-cure resin cement was then immediately evaluated post-irradiation, with this condition designated as the “Immediate group”. Subsequently, the photopolymerized dual-cure resin cement was aged in an incubator at 37 °C for 24 h under light shielding, referred to as the “Aging group”. The resulting dual-cure resin cements were characterized based on their degree of conversion and Vickers hardness in subsequent experiments.

### 2.3. Fourier Transform Infrared (FTIR) Spectroscopy

**Figure 3:**
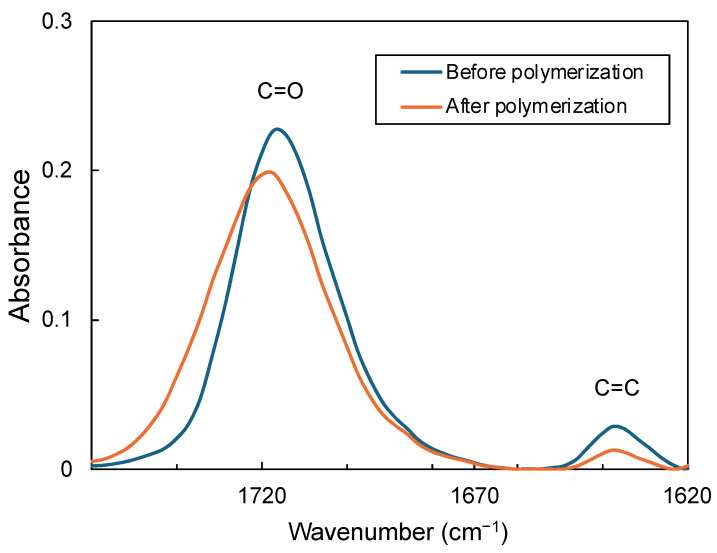
Typical FTIR spectra of the dual-cure resin cements before and after polymerization.

The polymerization of the dual-cure resin cements for each experimental group was characterized by degree of conversion, which was estimated using FTIR spectroscopy. FTIR measurements were performed using an FTIR spectrometer (IRSpirit; Shimadzu Corp., Kyoto, Japan) equipped with an attenuated total reflectance unit (QATR-S; Shimadzu Corp.). In the FTIR spectra, two characteristic bands at 1638 cm^−1^ (C=C stretch vibration) and 1720 cm^−1^ (C=O stretch vibration) before and after polymerization of the dual-cure resin cement were used to calculate the degree of conversion using the following equation [22,23]:

(1) $$Degree\;of\;conversion(\%)=\left(1-\frac{{\left[A(C=C)/A(C=O)\right]}_{before\;polymerization}}{{\left[A(C=C)/A(C=O)\right]}_{after\;polymerization}}\right)\times 100$$

where A(C=C) and A(C=O) are the absorbance values. Five samples were used to measure the degree of conversion in each group (n = 5). Figure 3 illustrates an example of the FTIR spectra for the dual-cure resin cements, illustrating the changes before and after photopolymerization.

### 2.4. Microhardness Test

After the FTIR measurements, the samples were subjected to Vickers microhardness tests to determine the mechanical properties of the cement in each experimental group. For each group, a microhardness test was performed using a Vickers hardness tester (HMV-G21ST, Shimadzu Corp., Kyoto, Japan) with a load of 50–200 g and a dwell time of 15 s. Five samples were used to measure the Vickers hardness for each group (n = 5).

### 2.5. Statistical Analysis

The obtained data were analyzed using a statistical software program (EZR version 1.62, Saitama Medical Center, Jichi Medical University, Saitama, Japan), with one-way variance (ANOVA) followed by Tukey’s post-hoc test, to compare statistical differences between the groups. Statistical significance was set to (*p*) < 0.05 for all analyses. The independent samples Student’s *t*-test was used to compare the values of the immediate and aged samples for each group.

## 3. Results

### 3.1. Degree of Conversion

**Figure 4:**
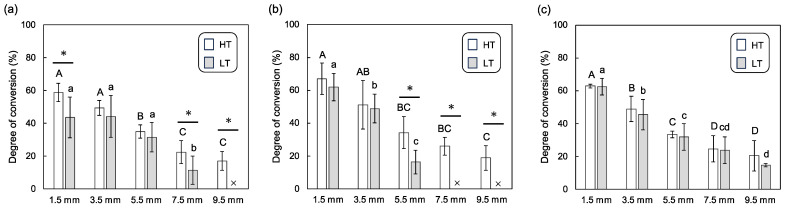
Mean and standard deviation for degree of conversion (%) for the dual-cure resin cements in immediate groups, photopolymerized through different thicknesses of CAD/CAM material plates; (**a**) lithium disilicate glass, (**b**) resin composite, and (**c**) polymer-infiltrated ceramic network with high-translucency (HT) and low-translucency (LT) grades. The samples that did not cure at all are indicated with “×” symbols. Different uppercase and lowercase letters represent statistically significant differences among different plate thicknesses within HT or LT groups, respectively. An asterisk indicates a statistically significant difference between the HT and LT groups within the same plate thickness.

Figure 4 shows the degree of conversion of dual-cure resin cement in the immediate group. In the case of lithium disilicate glass (Figure 4a), the degree of conversion decreased with increasing material thickness. For thicknesses of 1.5, 7.5, and 9.5 mm, the degree of conversion in the HT group was statistically higher than that in the LT group. For the resin composite (Figure 4b), a similar trend of decreasing degree of conversion with increasing plate thickness was observed. There were statistically significant differences between the degree of conversion for the HT and LT groups at plate thicknesses of 5.5, 7.5, and 9.5 mm. For the polymer-infiltrated ceramic network (Figure 4c), the degree of conversion also diminished with increasing plate thickness. When comparing the HT and LT groups, no significant differences were found in the degree of conversion across various plate thicknesses.

**Figure 5:**
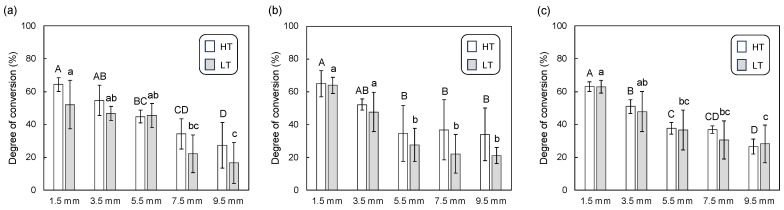
Mean and standard deviation for degree of conversion (%) for the dual-cure resin cements in aging groups, photopolymerized through different thicknesses of CAD/CAM material plates; (**a**) lithium disilicate glass, (**b**) resin composite, and (**c**) polymer-infiltrated ceramic network with high-translucency and low-translucency grades. Different uppercase and lowercase letters represent statistically significant differences among different plate thicknesses within HT or LT groups, respectively.

Figure 5 shows the degree of conversion of the dual-cure resin cement in the aging groups. Across all CAD/CAM materials, the degree of conversion decreased as the plate thickness increased. In this instance, no statistical difference was observed between the HT and LT groups across plate thicknesses for each CAD/CAM material.

### 3.2. Vickers Hardness

**Figure 6:**
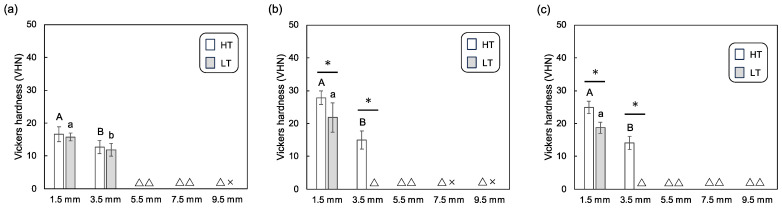
Mean and standard deviation for Vickers hardness for the dual-cure resin cements in immediate groups, photopolymerized through different thicknesses of CAD/CAM material plates; (**a**) lithium disilicate glass, (**b**) resin composite, and (**c**) polymer-infiltrated ceramic network with high-translucency (HT) and low-translucency (LT) grades. In the figures, the cement samples that were cured but remained too soft for Vickers hardness measurement are denoted with “△” symbols. The samples that did not cure at all are indicated with “×” symbols. Different uppercase and lowercase letters represent statistically significant differences among different plate thicknesses within HT or LT groups, respectively. An asterisk indicates a statistically significant difference between the HT and LT groups within the same plate thickness.

Figure 6 shows the Vickers hardness of the dual-cure resin cements in the immediate group. For the lithium disilicate glass (Figure 6a), the Vickers hardness decreased with increasing plate thickness. At thicknesses of 5.5 and 7.5 mm, the Vickers hardness could not be measured as the dual-cure resin cement was too soft, falling below the lower measurement limit. At a 9.5 mm thickness in the LT group, the dual-cure resin cement could not be cured at all. In the case of the resin composite (Figure 6b), a similar trend was observed with a decrease in the Vickers hardness as the plate thickness increased. For thicknesses of 5.5, 7.5, and 9.5 mm, Vickers hardness measurements were not possible because of the excessive softness of the dual-cure resin cement. For the polymer-infiltrated ceramic network (Figure 6c), the Vickers hardness decreased with increasing plate thickness. Similar to the resin composite, the Vickers hardness for the thicknesses of 5.5, 7.5, and 9.5 mm could not be measured because the dual-cure resin cement was not sufficiently cured to allow measurement.

**Figure 7:**
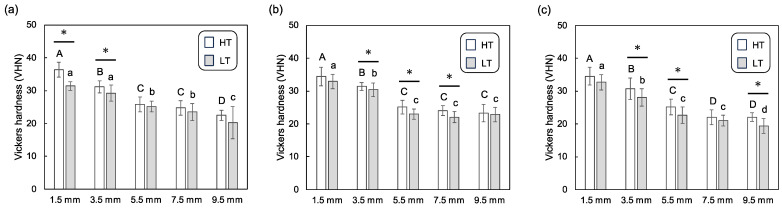
Mean and standard deviation for Vickers hardness for the dual-cure resin cements in aging groups, photopolymerized through different thicknesses of CAD/CAM material plates; (**a**) lithium disilicate glass, (**b**) resin composite, and (**c**) polymer-infiltrated ceramic network with high-translucency (HT) and low-translucency (LT) grades. Different uppercase and lowercase letters represent statistically significant differences among different plate thicknesses within HT or LT groups, respectively. An asterisk indicates a statistically significant difference between the HT and LT groups within the same plate thickness.

Figure 7 shows the Vickers hardness of the dual-cure resin cements in the aging groups. Across all CAD/CAM materials, a decrease in the Vickers hardness was observed with increasing cement thickness. Unlike the immediate groups, all aging samples were sufficiently cured, allowing their Vickers hardness to be measured. Statistical differences were observed between the HT and LT groups for the 1.5 and 3.5 mm thicknesses for the lithium disilicate glass; for the 3.5, 5.5, and 7.5 mm thicknesses for the resin composite; for the 3.5, 5.5, and 9.5 mm thicknesses for the polymer-infiltrated ceramic network, respectively.

## 4. Discussion

The objective of this study was to elucidate the effects of the CAD/CAM material thickness and translucency grade (high- or low-translucency) on the polymerization process of dual-cure resin cement. The polymerization of dual-cure resin cements, both immediately after light irradiation and after aging for 24 h in addition to light irradiation, was characterized by the degree of conversion and Vickers hardness. The findings revealed that both the degree of conversion and Vickers hardness decreased as the thickness increased for all the examined CAD/CAM materials, namely, lithium disilicate glass, resin composite, and polymer-infiltrated ceramic networks. Consequently, the first null hypothesis, which posited that the thickness of the CAD/CAM materials does not influence the polymerization of the dual-cure resin cement, is rejected. Moreover, the results demonstrated that the translucency grade of each CAD/CAM material affected both the degree of conversion and Vickers hardness in the immediate groups, whereas it had no effect on the degree of conversion and little effect on the Vickers hardness in the aging groups. Therefore, the second null hypothesis, which stated that the translucency grades of CAD/CAM materials do not affect the polymerization of dual-cure resin cement, is partially rejected.

In this study, two methods were employed to characterize the polymerization behavior of dual-cure resin cement: degree of conversion and Vickers hardness. The results, as estimated by both degree of conversion and Vickers hardness, exhibited a consistent trend: an increase in the material thickness led to a decrease in both the Vickers hardness and degree of conversion. These methods effectively verified the polymerization behavior of dual-cure resin cement. Previous studies on the polymerization of resin-based materials have shown that polymerization behavior can be similarly estimated using the degree of conversion and/or hardness measurements [24,25,26]. The degree of conversion can be determined using FTIR spectroscopy [27] and Raman spectroscopy [28,29]. Several methods exist for measuring the hardness of resin-based materials, including the Vickers [30,31], Knoop [24,29], and Martens hardnesses [32,33]. However, it is important to note that hardness measurements may not always be feasible, particularly if the dual-cure resin cement is too soft. In some instances, the dual-cure resin cement appeared to have solidified; however, its hardness could not be measured. In contrast, the degree of conversion was obtainable for such samples. Therefore, it is advisable to use both the hardness and degree of conversion assessments, rather than evaluating only one, to corroborate each set of results.

For endocrown restorations, CAD/CAM materials are favored because of their durability, aesthetic qualities, and adhesive bonding abilities [1,34,35]. Ceramics are the preferred materials for endocrowns, with clinical reports citing the use of lithium disilicate glass [4], zirconia [36], leucite-reinforced ceramic [37], and feldspar porcelain [38]. Among these, lithium disilicate glass is often considered the most suitable for endocrowns because of its excellent bonding ability and mechanical durability [34]. In addition to ceramics, resin composites are utilized for endocrown fabrication [39]. Their lower brittleness compared to traditional ceramics makes them a viable choice for endocrowns, particularly in patients with bruxism or in those requiring restoration to withstand heavier occlusal forces. Polymer-infiltrated ceramic networks represent a unique category of hybrid materials that combine the advantages of ceramics and polymers. This material has mechanical properties that are highly compatible with human teeth [40]. Owing to their balanced mechanical and physicochemical properties, polymer-infiltrated ceramic networks are increasingly being recognized as a suitable choice for endocrown applications [41]. These CAD/CAM materials for endocrowns are available in HT and LT grades and offer different levels of optical translucency to achieve aesthetically pleasing tooth restorations. Previous studies have investigated the impact of ceramic translucency on the polymerization of resin-based cements by the light irradiation of ceramic [21,42,43]. Kuijper et al. [21] examined the influence of lithium disilicate translucency at thicknesses of 4.0 mm and 7.5 mm on the degree of conversion of dual-cure resin cement. Their findings suggested that the translucency grade of the lithium disilicate glass did not affect the degree of conversion. Similarly, Chen et al. [42] explored the effect of lithium disilicate glass translucency on the polymerization of dual-cure resin cement through 1 mm- and 2 mm-thick samples, finding no significant effect on the microhardness or degree of conversion. In line with these findings, the present study revealed that differences in the translucency of all the examined CAD/CAM materials affected the polymerization of the dual-cure resin cement immediately after light irradiation, with little or no influence on polymerization after aging. Based on the results of these studies, the translucency grade of the material has little influence on the polymerization of dual-cure resin cement in endocrown restorations.

The experimental results of this study demonstrate that the polymerization of dual-cure resin cement diminishes with increasing material thickness. These findings are particularly relevant when comparing endocrowns with conventional crowns. For conventional crowns, a material thickness of 1–2 mm is typical [44,45]. In contrast, endocrowns are characterized by greater thicknesses, generally exceeding 4.0 mm [46,47,48,49]. This is due to the large combined thickness of the crown (at least 2.0 mm) and pulp chamber extension (over 2 mm), with the overall thickness exceeding 4.0 mm for endocrowns. In this study, distinct differences were observed in the degree of conversion and Vickers hardness between the groups with 1.5 mm thickness (conventional crowns) and those with 5.5 mm or greater thickness (endocrown).

Dual-cure resin cement is an adhesive widely used in various dental restorative procedures. The term “dual-cure” reflects its dual mechanism of hardening or setting: through light curing and self-curing. Light curing of the cement occurs immediately upon exposure to light irradiation, whereas self-curing occurs gradually when the two components of the cement are mixed. This dual mechanism is particularly beneficial in areas where light cannot adequately penetrate. Given the substantial thickness of the endocrowns, light penetration into the cement is often limited, thereby diminishing the efficacy of light curing. In such scenarios, the self-curing property of dual-cure resin cement becomes crucial. The findings of this study reveal a notable observation: the degree of polymerization and Vickers hardness were significantly higher in samples with HT grade compared to those with LT grade in the initial group. However, this difference diminished in the aging group, highlighting the crucial influence of the self-curing properties of the dual-cure resin cement. This phenomenon underscores the adaptability of dual-cure resin cements in dental restorations, especially for applications involving substantial material thickness or complex geometries that hinder light accessibility. The self-curing mechanism ensures a dependable setting process throughout the material, compensating for areas not reached by light. It emphasizes the importance of considering both curing methods when assessing the performance and long-term stability of dental restorations.

Our experiments revealed that cement polymerization was insufficient with light irradiation alone. Meanwhile, post-polymerization occurred over time, leading to an increase in both the degree of conversion and Vickers hardness. Despite post-polymerization involving aging, the degree of conversion and Vickers hardness for a material thickness of 5.5 mm were not as high as those observed for a thickness of 1.5 mm. Consequently, the polymerization of dual-cure resin cement in endocrowns is less efficient than that in conventional crowns, even after considering the post-polymerization effects of self-curing. This result suggests that the polymerization of dual-cure resin cement in endocrown restorations may be inferior to that in conventional crown restorations. Consequently, when using dual-cure resin cement to bond endocrowns to abutment teeth, there is a potential risk of insufficient polymerization of the dual-cure resin cement, which could lead to clinical failures such as debonding or fracture. Clinical reports on endocrowns have indicated a higher incidence of debonding failures, particularly in cases where dual-cure or light-cure resin cements are used as adhesives [11,50]. The choice of Panavia V5 as the dual-cure resin cement for this study leverages its well-documented success in bonding CAD/CAM materials [51], making it a representative candidate for examining the effectiveness of dual-cure resin cements in such applications. Nevertheless, it is crucial to acknowledge the diversity of polymerization behaviors among the various dual-cure resin cements available commercially [52]. This variability suggests that the performance of endocrown bonding could be significantly influenced by the distinct properties inherent to each brand of resin cement.

Although informative, this study has several limitations and does not entirely replicate the clinical application of endocrowns. One significant limitation was the use of material in plate form, which failed to accurately mimic the complex tooth shape typical of endocrowns. Additionally, the specific orientation of the light irradiation through the endocrown to the dual-cure resin cement was not considered in this experimental setup. The intensity and duration of light irradiation are known to significantly influence cement polymerization. In our experiment, we employed a relatively high irradiation intensity and extended the irradiation time to ensure effective curing of the cement. However, the dependence of polymerization on the irradiation intensity and duration warrants further investigation. In addition, this study did not utilize human teeth, opting instead for a simplified experimental approach. To bridge the gap between experimental settings and clinical practice, future studies should include experiments that consider these critical factors. This includes both in vitro and in vivo studies designed to closely align with the complexities and variables encountered in dental treatments.

## 5. Conclusions

This study aimed to elucidate the polymerization behavior of dual-cure resin cement in endocrowns by examining the degree of conversion and Vickers hardness after curing with varying thicknesses of CAD/CAM materials (lithium disilicate glass, resin composite, and polymer-infiltrated ceramic network), in both high- and low-translucency grades. Considering the limitations of this study, the following conclusions were drawn.

- Polymerization of the dual-cure resin cement decreased with increasing CAD/CAM material thickness, suggesting that thicker materials impeded the curing process.
- Significant differences in polymerization were observed between samples with a 1.5 mm thickness (conventional crowns) and those with a thickness of 5.5 mm or greater (endocrowns).
- The translucency grades influenced the polymerization of the dual-cure resin cement immediately after light irradiation. However, these differences were either eliminated or substantially reduced after post-polymerization treatment by aging.

These findings suggest that when dual-cure resin cements are employed in endocrowns, the degree of polymerization and Vickers hardness may be insufficient compared with conventional crowns, irrespective of the translucency grade and thickness of the CAD/CAM material.

## Figures and Tables

**Table 1:** CAD/CAM material blocks used in this study. The material composition is based on the information published by the manufacturer.

Material Type	Product Name	Product Company	Composition
Lithium disilicate glass	IPS e.max CAD	Ivoclar Vivadent, Schaan, Liechtenstein	SiO_2_, Li_2_O, K_2_O, P_2_O_5_, ZrO_2_, ZnO, Al_2_O_3_, MgO, Coloring oxide
Resin composite	CERASMART300	GC Corp., Tokyo, Japan	SiO_2_, Barium glass, urethane dimethacrylate, 2,2-bis(4-methacryloxypolyethoxyphenyl)propane
Polymer-infiltrated ceramic network	VITA ENAMIC	VITA Zahnfabrik, Nabertherm, Germany	SiO_2_, A_l2_O_3_, Polymer, Na_2_O, K_2_O, B_2_O_3_, ZrO_2_, CaO

## Data Availability

Data are contained within article.
